# Computational Fluid Dynamics for the Prediction of Endograft Thrombosis in the Superficial Femoral Artery

**DOI:** 10.1177/15266028221091890

**Published:** 2022-04-25

**Authors:** Lennart van de Velde, Erik Groot Jebbink, Rob Hagmeijer, Michel Versluis, Michel M. P. J. Reijnen

**Affiliations:** 1 Department of Surgery, Ziekenhuis Rijnstate, Arnhem, The Netherlands; 2 Multi-Modality Medical Imaging Group, Technical Medical Centre, University of Twente, Enschede, The Netherlands; 3 Physics of Fluids Group, Technical Medical Centre, University of Twente, Enschede, The Netherlands; 4 Engineering Fluid Dynamics, University of Twente, Enschede, The Netherlands

**Keywords:** computational flow dynamics, peripheral artery disease, stent thrombosis, hemodynamics, wall shear stress

## Abstract

**Purpose:**

Contemporary diagnostic modalities, including contrast-enhanced computed tomography (CTA) and duplex ultrasound, have been insufficiently able to predict endograft thrombosis. This study introduces an implementation of image-based computational fluid dynamics (CFD), by exemplification with 4 patients treated with an endograft for occlusive disease of the superficial femoral artery (SFA). The potential of personalized CFD for predicting endograft thrombosis is investigated.

**Materials and Methods:**

Four patients treated with endografts for an occluded SFA were retrospectively included. CFD simulations, based on CTA and duplex ultrasound, were compared for patients with and without endograft thrombosis to investigate potential flow-related causes of endograft thrombosis. Time-averaged wall shear stress (TAWSS) was computed, which highlights areas of prolonged residence times of coagulation factors in the graft.

**Results:**

CFD simulations demonstrated normal TAWSS (>0.4 Pa) in the SFA for cases 1 and 2, but low levels of TAWSS (<0.4 Pa) in cases 3 and 4, respectively. Primary patency was achieved in cases 1 and 2 for over 2 year follow-up. Cases 3 and 4 were complicated by recurrent endograft thrombosis.

**Conclusion:**

The presence of a low TAWSS was associated with recurrent endograft thrombosis in subjects with otherwise normal anatomic and ultrasound assessment and a good distal run-off.

## Introduction

For complex above-the-knee femoropopliteal artery occlusive disease, heparin-bonded covered endografts have shown superior patency rates compared to bare metal stents^1^ and exhibit 1 year patency rates comparable to bypass surgery.^2^ Still, with reported 3 year primary and secondary patency rates for heparin-bonded endografts of 59% and 82%, respectively, complications and reinterventions are not uncommon.^3,4^ Endograft thrombosis in particular may have a major impact as it can cause a compartment syndrome after revascularization or limb loss. The development of an edge stenosis that limits and/or disturbs blood flow through the endograft is commonly considered as an important risk factor for endograft thrombosis. This is supported by observations that an edge stenosis was present in 75% of thrombosed endografts,^5^ mostly at the proximal edge of the endograft. Although the introduction of a contoured proximal graft edge and the strategy of minimizing oversizing might reduce the incidence of edge stenosis, edge stenosis is still a common complication in treated patients and may predispose to thrombosis.^6^

A contributor to the risk on the development of a proximal edge stenosis and endograft thrombosis may be the proximity of the endograft to the femoral bifurcation. The flow division at the bifurcation may generate focal areas of slow and recirculating flow that, if persistent over the cardiac cycle, are likely to promote anastomotic intimal hyperplasia or thrombus formation.^7,8^ These areas of stasis of blood are undetectable by current clinical imaging modalities including duplex ultrasound and contrast-enhanced computed tomography (CTA). Imaging-based computational fluid dynamics (CFD) can compute the complete 3D flow field over time in patient geometries and therefore has potential value for diagnosing these areas of slow and recirculating flow in patients. Computational fluid dynamics has a proven track record for diagnosing the hemodynamic significance of coronary artery disease.^9^ For arterial thrombosis, CFD has been applied for understanding thrombosis for bare metal stents in coronary arteries^10^ and carotid arteries,^11^ as well as for exploring hemodynamic causes of intraluminal thrombus in cerebral aneurysms^12^ and aortic aneurysms.^13^ In addition, a few hypothesis-generating studies for endograft thrombosis in stented carotid,^14^ aortic,^15,16^ and femoropopliteal^17^ arteries have been described. However, most of the studies on stented arteries have not correlated the hemodynamic simulations with longitudinal patient follow-up. Moreover, none of these studies have assessed the value of CFD for predicting endograft thrombosis, as none included a set of control cases in which no thrombosis occurred. Here we demonstrate the clinical feasibility of patient-specific CFD in 4 patients treated with an endograft in the superficial femoral artery (SFA), of which half experienced endograft thrombosis on follow-up. By comparing simulation results for patients with and without endograft thrombosis, the value of CFD for predicting endograft complications is investigated.

## Materials and Methods

### Clinical Cases

**Figure 1.**
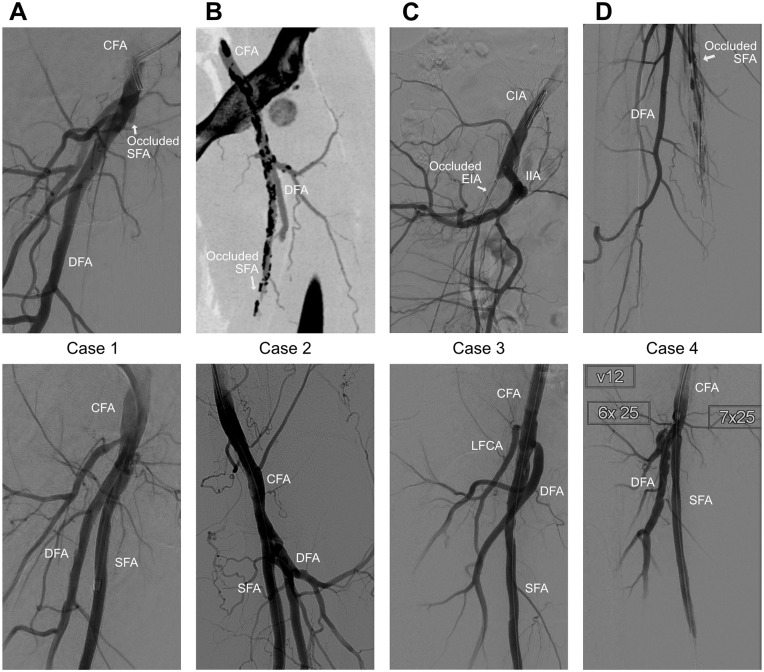
Digital subtraction angiography (DSA) images before (top) and after (bottom) stent graft placement in the SFA. For case 2, a preprocedural CTA-slice is shown, as a DSA before the stent placement was not available. Similarly for case 3, a DSA of the occluded SFA was not available, so a DSA of the occluded external iliac artery is instead shown. CFA, common femoral artery; CIA, common iliac artery; DFA, deep femoral artery; EIA, external iliac artery; IIA, internal iliac artery; LFCA, lateral femoral circumflex artery; SFA, superficial femoral artery.

Four patients were selected with a successful recanalization of an occluded SFA by the placement of an expanded polytetrafluoroethylene endograft (Heparin-bonded Viabahn; W.L. Gore & Associates, Flagstaff, AZ, USA). In 3 of these patients, an additional thromboendarterectomy of the common femoral artery (CFA) was performed. In all cases, the proximal end of the endograft was near the femoral bifurcation (<3 cm). The indication for intervention was Rutherford 3 (n=1), 4 (n=1), and 6 (n=2), respectively. Dual anti-platelet (acetylsalicylic acid 80 mg 1 dd and clopidogrel 75 mg 1 dd) was prescribed for 3 months followed by a single antiplatelet regimen. Patient characteristics and procedural details are provided in Table 1 and procedural images are shown in Figure 1. This study was approved by the competent institutional review board and written informed consent for data publication was provided by all participants.

**Table 1.** Patient Characteristics and Procedural Data.

	Case 1	Case 2	Case 3	Case 4
Age (years)	77	78	63	67
Gender	Male	Male	Female	Male
Rutherford	6	3	6	4
Comorbidities	DMII, diabetic foot syndrome, heart failure	DMII, CABG, hypercholesteremia, hypertension		DMII, MI
Intoxications	Active smoker	Past smoker	Past smoker	Active smoker until third occlusion
Hematocrit	0.43	0.36	0.3	0.35
Treated side	Right	Left	Right	Right
Endograft sizing (mm)	Viabahn 5x250 + 5x250	Viabahn 6x250 + 7x150	Viabahn 6x250 Viabahn 8x100 (ipsilateral EIA)	Viabahn 6x250, + 7x40 at second thrombolysis
Concomitant procedures	Thromboendarterectomy with patch angioplasty of the CFA	Thromboendarterectomy with patch angioplasty of the CFA	Thromboendarterectomy with patch angioplasty of the CFA and proximal SFA	DCB popliteal + PBA PTA, TPT and peroneal artery
Number of patent run-off vessels	3	3	3	2
Time of CTA (months)	15M	6M	29M	11M
Secondary procedures	7M: DCB of popliteal stenosis	4M: PBA of progressive stenosis CFA 11M: DCB of restenosis CFA 20M: PBA of in-stent stenosis	8M: thrombolysis of occlusion SFA and PBA inflow stenosis 28M: thrombolysis of second occlusion SFA and DCB inflow stenosis; compartment syndrome	4M: thrombolysis of occlusion SFA 7M: thrombolysis for second occlusion and proximal extension Viabahn 10M: thrombolysis for third occlusion

Abbreviations: CABG, coronary artery bypass graft; DMII, Diabetes mellitus II; DCB, drug coated balloon; EIA, external iliac artery; M, months; MI, myocardial infarction; PBA, plain balloon angioplasty; PTA, posterior tibial artery; TPT, tibioperoneal trunk.

### Computational Fluid Dynamics

Physical flow equations of 3-dimensional blood flow were solved with patient-specific CFD simulations. The CFD model employed the patient’s anatomy segmented from CTA and duplex ultrasound measurements for the boundary conditions, as illustrated in Supplemental Figure 1.

A CTA with 0.9 mm slice thickness (IQon spectral CT with iDose reconstruction, Philips Healthcare, Amsterdam, the Netherlands) was used for reconstruction of the geometry. For segmentation and smoothing of the arterial lumen, the vmtkLab software (v1.6.1, Orobix, Bergamo, Italy) employing a level-set technique based on implicit active surfaces^18^ was used. This segmentation technique utilizes a deformable 3D-geometry that is simultaneously deformed to conform to user-weighted constraints on attraction to the vessel lumen boundary and surface model curvature. Only high-level choices such as vessel selection, a single lumen boundary weight and a single curvature weight per vessel can be made by the user, which provides a high inter- and intraoperator repeatability of the segmentation.^19,20^ The 3 major femoral arteries were segmented, without inclusion of the lateral circumflex artery stemming from the deep femoral artery (DFA).

To derive the inlet and outlet boundary conditions, the centerline velocities measured with duplex ultrasound at the CFA, proximal DFA and mid SFA were used to compute time-varying flow rates by assuming a fully developed Womersley profile, which provides an explicit relation between centerline velocity and flow rate.^21^ For the DFA, the peak velocity was measured relatively close to the femoral bifurcation, which could have led to overestimation of total volume flow here by the fully developed flow assumption. The measured CFA flow profile was imposed at the inlet as a Womersley velocity profile and a 3-element Windkessel was applied at the SFA and DFA outlets, tuned to match the measured flow profiles. The values obtained after tuning the Windkessel outlets are shown in Supplementary Table 1.

The timing of the CTA and hematocrit levels for the CFD simulation are depicted in Table 1, with further details on modeling choices and the SimVascular CFD solver^22^ in the expanded methods section. The CFD solver has been validated against phase-contrast magnetic resonance imaging (PC-MRI) in realistic patient geometries.^23^ From the CFD solution, the time-averaged wall shear stress (TAWSS) over 1 cardiac cycle was calculated. Areas of low TAWSS correspond to sites of recirculating flow, and a TAWSS < 0.4 Pa has been associated with proatherogenic endothelial activity.^24^ In the context of graft thrombosis, low TAWSS translates to a decreased washout of particles, leading to prolonged residence times of blood particles. In vitro studies have demonstrated the preferential accumulation of fibrin networks at sites of low TAWSS.^25^ The full modeling process, including segmentation and postprocessing, could be completed in approximately 7 hours, consisting of 3 hour human labor and 4 hour computational time (~45 minutes per cardiac cycle) on an entry-level 64 core computational cluster (4 × 16 core Opteron 6376, Advanced Micro Devices, Santa Clara, CA, USA). Segmentation was the most time-intensive process of the human labor, in part because a computationally expensive segmentation method was used to meet the stringent requirements CFD puts on continuity and smoothness of the geometry. This method can likely be significantly sped up with numerical optimizations.

## Results

### Clinical Follow-Up

**Figure 2.**
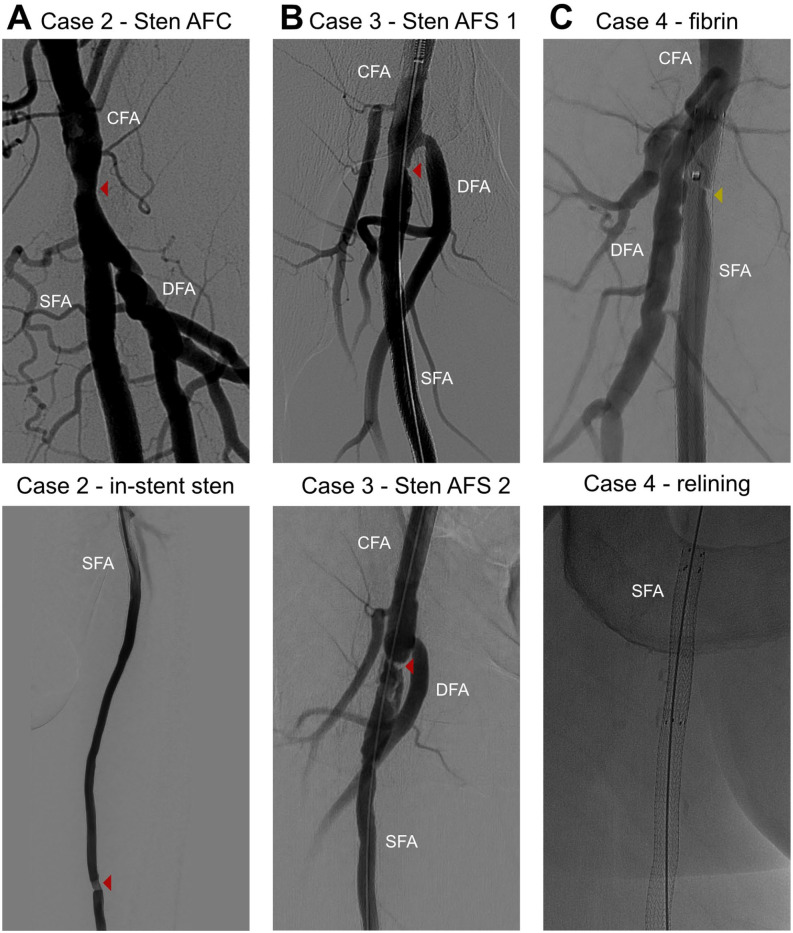
DSA-images for a selection of secondary treatments. All marked lesions were treated with PTA, with the addition of a drug-coated balloon for the recurring inflow stenosis in the SFA (B bottom). An area devoid of contrast was found in the proximal Viabahn for case 4 (C top), attributed to fibrin deposition, treated with PTA and a proximal extension with a second Viabahn stent graft (C bottom). CFA, common femoral artery; DFA, deep femoral artery; PTA, posterior tibial artery; SFA, superficial femoral artery.

All patients had a good short-term follow-up, with no abnormalities observed on 6 week duplex ultrasound. Afterward, case 1 developed a popliteal stenosis ~8 cm distal from the distal edge of the endograft, with a peak systolic velocity ratio > 6 that was treated at 7 months using plain balloon angioplasty (PBA) and a drug-coated balloon (DCB) (IN.PACT Admiral, Medtronic, Minneapolis, MN). Thereafter the follow-up was uneventful up to the latest follow-up at 26 months. Case 2 was characterized by a progressive stenosis in the CFA, which was treated by PBA at 4 month follow-up (Figure 2A). A CTA was made at 7 months follow-up for planning of a thromboendarterectomy of the contralateral CFA. The ipsilateral CFA restenosis resurfaced at 11 months follow-up, which was treated for a second time with PBA. At 20 month follow-up, an ipsilateral in-stent stenosis >50% was observed on duplex ultrasound (Figure 2A) and treated with PBA. No further complications occurred up to the patient’s death at 41 months.

Patient 3 presented twice with an acute occlusion of the endograft at 8 and 28 months, respectively. Both were successfully treated with catheter-directed thrombolysis, although the second treatment was complicated by a compartment syndrome treated with fasciotomy. During the first thrombolysis, a stenosis near the proximal edge of the endograft was observed (Figure 2B) and treated with PBA. In addition, antithrombotic medication was temporarily changed to coumadin, which was reverted to single antiplatelet therapy after 6 months. The inflow stenosis resurfaced at the second thrombolysis (Figure 2B) and was treated with a DCB. Antithrombotic medication was switched indefinitely to coumadin thereafter. To identify other potential causes of the recurring occlusion, the distal vasculature was evaluated with CTA and duplex ultrasound, both showing patency of the SFA without restenosis and a 3 vessel distal run-off. Nonetheless, routine duplex ultrasound follow-up demonstrated reocclusion of the endograft half a year later, which was left untreated in the absence of symptoms.

Patient 4 presented with a cold leg and sudden decrease in walking distance caused by an occlusion at 4, 7, and 10 months, all successfully mitigated by catheter-directed thrombolysis. After the first thrombolysis, the patient was switched from a single anti-platelet regimen to coumadin. During the second thrombolysis, a proximal region in the endograft devoid of contrast was found (Figure 2C), which did not fully resolve after PBA. Therefore, a 5 cm long second endograft was inserted in the first one up to the level of the femoral bifurcation (Figure 2C). This did not prevent a third occlusion, though. After the third thrombolysis, the endograft remained patent for the current 5 month follow-up period.

### Personalized Flow Simulations

**Figure 3.**
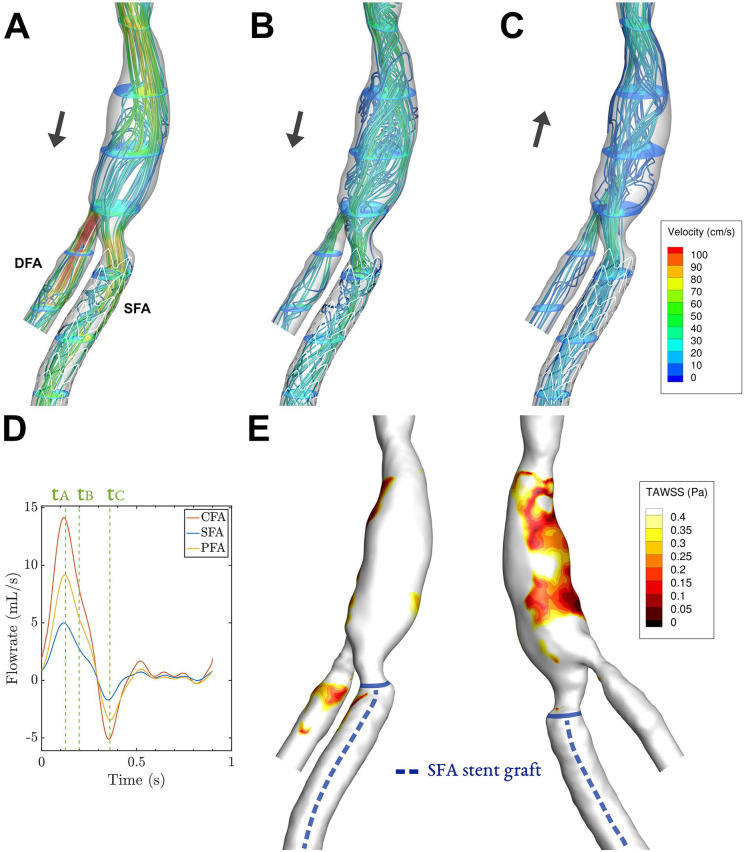
Case 1: Streamlines and velocity contours assessed by CFD for 3 key phases (A–C) of the cardiac cycle, corresponding to timepoints t_A_–t_C_ in in the cardiac cycle plot (D). The corresponding time-averaged wall shear stress (TAWSS) shows areas below 0.4 Pa in an anterior and posterior view (E). The black arrows in (A–C) depict whether net forward or backward flow is present. Video 1 shows the velocity vectors throughout the cardiac cycle. CFD, computational fluid dynamics; DFA, deep femoral artery, SFA, superficial femoral artery.

**Figure 4.**
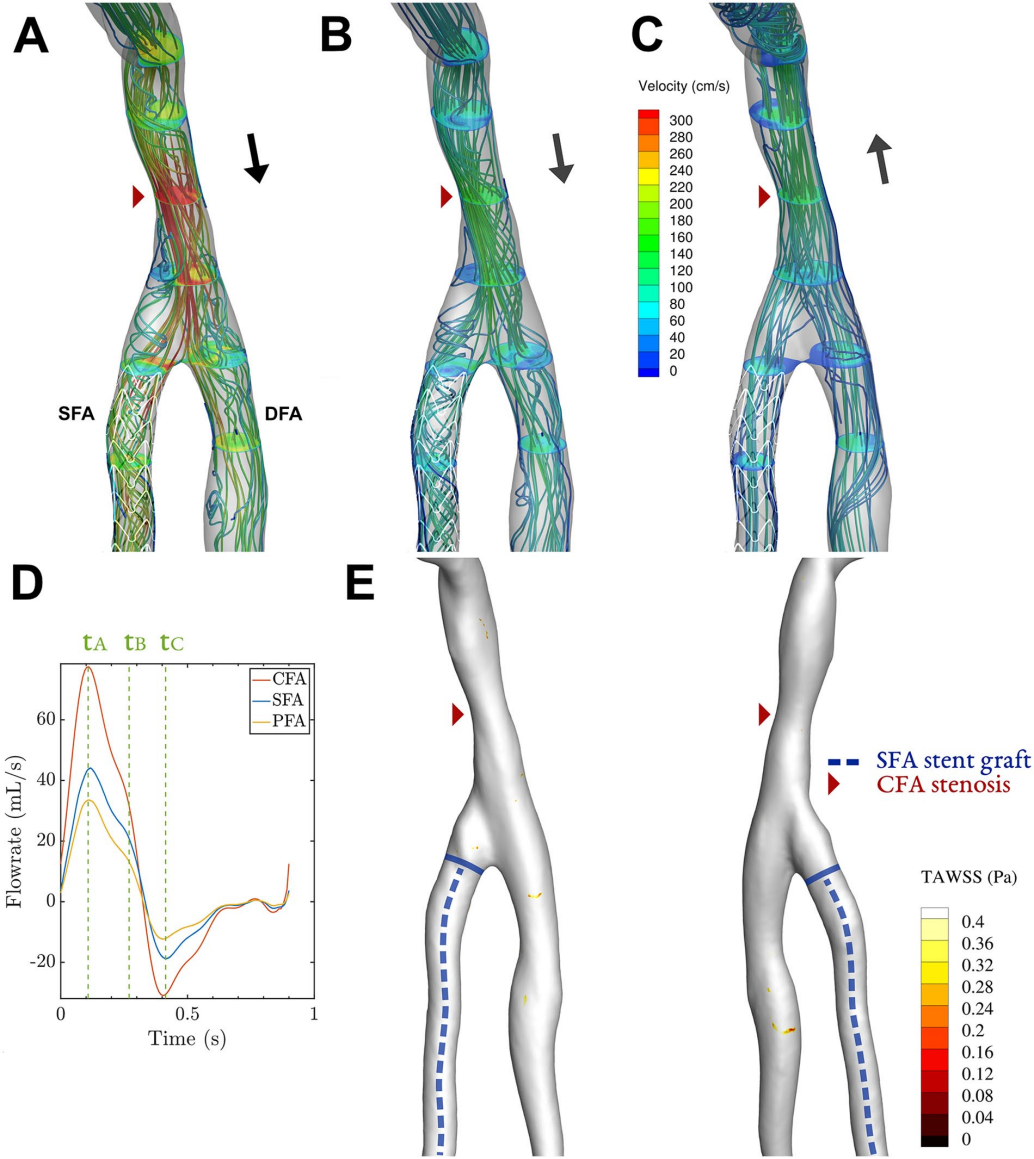
Case 2: Streamlines and velocity contours assessed by CFD for 3 key phases (A–C) of the cardiac cycle, corresponding to timepoints t_A_–t_C_ in in the cardiac cycle plot (D). The corresponding time-averaged wall shear stress (TAWSS) shows areas below 0.4 Pa in an anterior and posterior view (E). The black arrows in (A–C) depict whether net forward or backward flow is present. Video 2 shows the velocity vectors throughout the cardiac cycle. CFD, computational fluid dynamics; DFA, deep femoral artery, SFA, superficial femoral artery.

**Figure 5.**
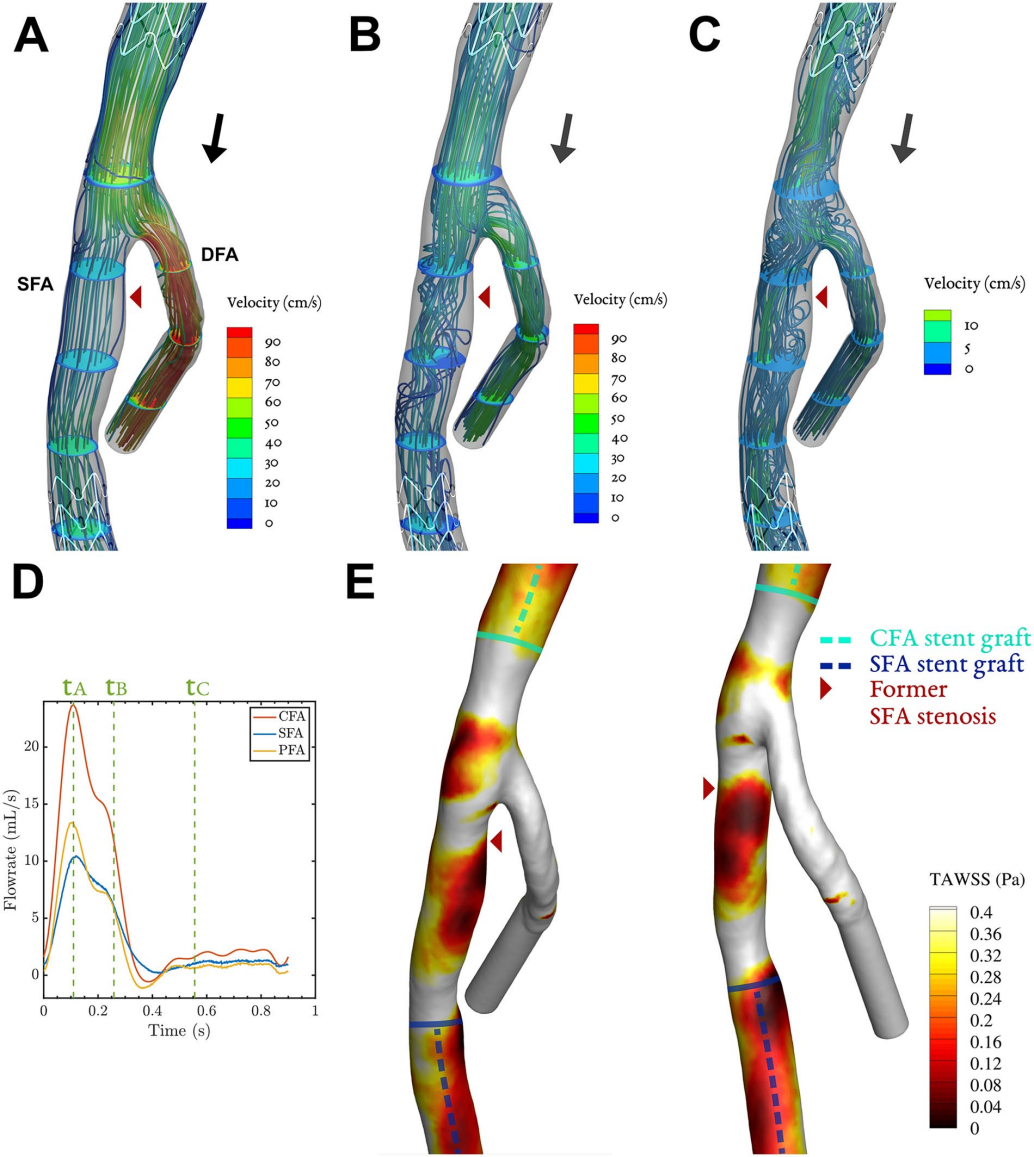
Case 3: Streamlines and velocity contours assessed by CFD for 3 key phases (A–C) of the cardiac cycle, corresponding to timepoints t_A_–t_C_ in in the cardiac cycle plot (D). The corresponding time-averaged wall shear stress (TAWSS) shows areas below 0.4 Pa in an anterior and posterior view (E). The black arrows in (A–C) depict whether net forward or backward flow is present. Video 3 shows the velocity vectors throughout the cardiac cycle. CFD, computational fluid dynamics; DFA, deep femoral artery, SFA, superficial femoral artery.

**Figure 6.**
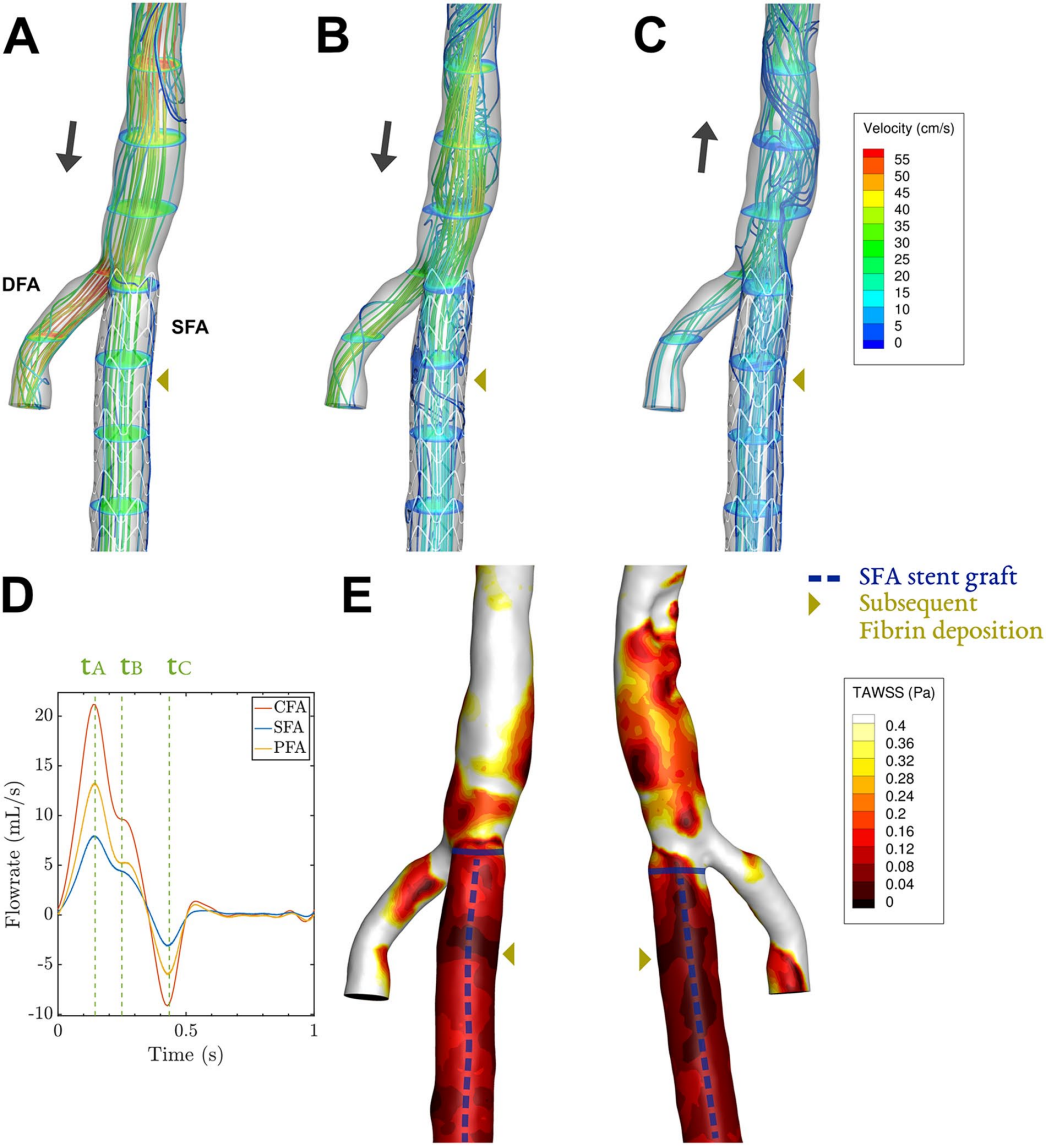
Case 4: Streamlines and velocity contours assessed by CFD for 3 key phases (A–C) of the cardiac cycle, corresponding to timepoints t_A_–t_C_ in in the cardiac cycle plot (D). The corresponding time-averaged wall shear stress (TAWSS) shows areas below 0.4 Pa in an anterior and posterior view (E). The black arrows in (A–C) depict whether net forward or backward flow is present. Video 4 shows the velocity vectors throughout the cardiac cycle. CFD, computational fluid dynamics; DFA, deep femoral artery, SFA, superficial femoral artery.

Instantaneous velocity streamlines for key phases of the cardiac cycle and TAWSS contours are shown in Figures 3–6 for all patients. Streamlines during the full cardiac cycle are animated in Videos 1–4. In all cases, flow separation was present most distinctively during systolic deceleration (timepoint tB). Case 1 was characterized by a widened CFA by patch angioplasty, which led to flow separation in the CFA. In addition, a slight narrowing at the origin of the SFA (and stent) was present, associated with modest flow separation in the SFA (Figure 3B). The flow separation in the CFA was associated with low TAWSS (<0.4 Pa, Figure 3E), which was not the case for the SFA where only a tiny streak of TAWSS < 0.4 Pa is present. Case 2 was characterized by a 50%-area stenosis in the CFA, creating high flow velocities and areas of helical or swirling flow, especially in the SFA (Figure 4A and B). The swirling flows with relatively high velocities were not associated with low TAWSS.

For case 3, flow separated in the SFA at multiple locations at the end of systole, in particular in a region encompassing the site of the recurrent inflow stenosis and at the endograft’s proximal edge. These zones with low flow velocity and recirculation were sustained during diastole (Figure 5C). Case 3 was the only case that exhibited monophasic flow on duplex ultrasound (Figure 5D), so no washout of low flow regions by backflow was present. Less consistent regions of flow separation were present more distal in the SFA endograft. The areas of flow separation in the SFA were associated with very low TAWSS, with values below 0.05 Pa (Figure 5E). These areas of minimal wall shear stress coincided with the location of the former inflow stenosis and the proximal edge of the endograft.

Similarly, for case 4, flow separation at multiple regions in the SFA was present, especially near the bifurcation (Figure 6B). Recirculating flow was also present in the CFA, which widens close to the bifurcation. Low values of TAWSS were seen at these sites, covering the full proximal SFA with values below 0.15 Pa (Figure 6E). The area in the proximal SFA devoid of contrast during the second thrombolysis, exhibited a zone of minimal TAWSS, with values below 0.01 Pa.

## Discussion

This hypothesis-generating study suggests that personalized CFD simulations can predict flow-induced complications in endografts in the SFA, where standard diagnostic imaging techniques failed to do so. The CFD simulations demonstrated pronounced areas of low TAWSS in the 2 cases that presented with a repetitive recurrent thrombosis of the endograft (patients 3 and 4). Areas of low TAWSS originated from zones of flow separation during systolic deceleration in cases 1, 3 and 4, which potentially associate with the lower velocities measured in the SFA, both in absolute terms and relative to DFA velocity. The flow separation generates adverse flow patterns, which can be stabilized by the backflow phase^26^ as in case 1, although this stabilization did not occur in case 4 despite similar triphasic flow. As the geometry and duplex-derived flow rates of case 1 and 4 were not very dissimilar, these cases demonstrate that the relevant flow features are difficult to derive from standard duplex and imaging data alone. Therefore, advanced techniques of flow characterization may offer clinical value for optimizing endograft treatment of occlusive disease of the SFA.

The underlying pathologic mechanism of endograft thrombosis is a complex multifactorial process, in which personalized CFD simulations could help unveil the contribution of flow separation, stasis of blood, and shearing forces. For this case of arterial coagulation, Virchow’s triad of surface susceptibility, hypercoagulability, and flow stasis likely all are important actors, possibly supplemented by shear-rate induced platelet activation.^27^ It is well documented that an endothelial layer does not form in surgically implanted ePTFE grafts in senescent humans, except for, at most, a ~1 cm region at either anastomosis. Likewise for ePTFE endografts, a stable endothelial layer does not seem to exceed 2 to 3 cm from the edges,^28^ with the remainder of the inner graft lining being covered by a layer of fibrin, mostly acellular, but possibly interspersed with platelets and granulocytes.^8^ It is unclear if these fibrin layers eventually develop on the heparin-bonded Viabahn, for which no histopathologic studies were found. Although the heparin-bonded luminal surface was shown to reduce thrombin activity in human blood,^29^ the graft surface remains more thrombogenic than endothelial-lined vessel walls.^30^ Over time, fibrin depositions may accumulate into macroscopic thrombus, especially at areas where residence time of blood particles is prolonged and where shear forces are small, that is, at sites of low TAWSS. This process can be accelerated by adverse flow patterns induced by an edge stenosis, which originates from anastomotic intimal hyperplasia at the graft edges.^8^

In the cases studied here, large areas with TAWSS below 0.15 Pa were present in the stent grafts that thrombosed (cases 3 and 4), but a small area with minima of 0.05 Pa was also present in one of the patent stents grafts (case 1). It is therefore difficult to postulate a precise cut-off level at which deposition occurs. A previous study has linked WSS below 0.17 Pa and below 0.41 Pa for a Newtonian fluid and for a shear-thinning viscosity model, respectively, to thrombus deposition in in-vitro flow loops.^31^ The cut-off for Newtonian CFD models at 0.17 Pa is of similar order as the adverse TAWSS levels observed in our cases. However, the shear-thinning cut-off at 0.41 Pa demonstrated a better fit to the experimental results in the mentioned study, but cannot be applied to our CFD results due to the assumed Newtonian behavior. Future computational studies of in-stent thrombosis may therefore benefit from using more complex viscosity models.

The hypothesized fibrin deposition at areas of low TAWSS may be especially relevant for endografts placed in the proximal SFA, as the underlying fluid mechanics predicts sites of flow separation in the region downstream of major bifurcations.^32^ The presence of such a pathophysiologic mechanism cannot be imaged by anatomic scanning or current duplex ultrasound techniques, but requires assessment of the 3D time-resolved vector flow field in endograft. At present, this can be accomplished by a costly and time-consuming 3D PC-MRI, by upcoming 3D ultrafast ultrasound techniques^33^ or by the present patient-specific CFD method, based on anatomic input by either CTA, MRI, or rotational angiography, and based on flow input for the 3 femoral arteries by duplex ultrasound. An advantage of CFD is that the effect of a treatment option on the persistence of adverse flow can be simulated, which could provide treatment decision support. Furthermore, material cost of CFD simulations is very low in comparison with other flow imaging techniques. Obviously, the suggested role of CFD in treatment decision support needs further clinical evidence of the hypothesized link between low TAWSS and endograft thrombosis.

To exemplify CFD treatment decision support for case 3, we investigated whether PBA of the luminal narrowing immediately upstream from the endograft could mitigate the low TAWSS region at the proximal stent edge. After virtual angioplasty of this vessel region by cylindrical inflation, CFD simulations with similar boundary conditions did not show a qualitative change of the localization of low TAWSS regions. However, at the proximal border of the endograft, the minimal value of TAWSS strongly increased from 0.015 to 0.1 Pa. When further substantiated by clinical evidence of the hypothesized association between TAWSS and endograft thrombosis, this could serve as an indication to perform PBA of the luminal narrowing to extend patency of the endograft. A more immediate implementation of CFD would be to personalize patient follow-up intervals and/or antithrombotic medication based on a risk stratification by TAWSS levels in and around the treated SFA.

Several limitations of the simulations and flow analysis warrant consideration in future studies. First, the used CTA images were performed at varying times during follow-up and do not accurately reflect the geometry after treatment. However, as all CTAs were made at 6M or later, the vascular geometry is not expected to change much as most vascular remodeling in response to treatment occurs within the first months.^8^ Second, the CFD simulations involve several simplifications and the accuracy of the simulations is dependent on the used CTA and duplex ultrasound input data, which were recorded in supine position and may be subject to change in other postural positions. The effect of the most important CFD modeling simplifications on WSS in bifurcating flows are a fully-developed velocity profile at the inlet (~10% change in TAWSS),^34^ a Newtonian behavior of blood (< 10% change in TAWSS),^35,36^ and a rigid wall assumption (~5% change in TAWSS).^37^ Although the absolute values of TAWSS in our simulations can be impacted by these simplifications, the referenced studies have shown that the localization of low TAWSS areas are not altered by these simplifications. Therefore, the low TAWSS values in our patient cases with occlusion relative to normal TAWSS values in control cases is a result that is likely not affected by these assumptions. As shown in a subset^34,36^ of these sensitivity studies, the major contributor to possible inaccuracies in the CFD results is uncertainty in CTA and duplex ultrasound input data. Main challenges for this study were segmentation of the endograft and the difficulty of obtaining strong duplex ultrasound signals in the DFA. The endograft segmentation resulted in a relatively smooth endograft inner surface, although it is known that some protrusions by the stent wires are present,^38^ which are not visible on CTA. These create some local fluctuations in TAWSS in the order of 0.05 Pa,^38^ which is small compared with the intra- and interpatient differences in our study. The impact of the suboptimal DFA duplex ultrasound signal is harder to assess and should be quantified in prospective clinical studies where duplex ultrasound data acquisition can be optimized for this specific purpose.

Finally, TAWSS is a somewhat surrogate measure of particle residence time and shearing forces, and more elaborate measures like wall shear stress exposure time and wall shear stress divergence^39^ may be better predictors of thrombus development. For reasonably laminar flow as present in the current cases, the differences are not as significant as for more complex transitional flows in abdominal aortic aneurysms, however.^39^ A prospective cohort study in a larger patient population with standardized image acquisition and follow-up would be ideally suited to address these limitations and to substantiate the hypothesized relation between wall shear stress and endograft thrombosis.

## Conclusion

Personalized CFD simulations demonstrated areas of low wall shear stress (TAWSS < 0.15 Pa) in the SFA endograft for 2 out of 4 patient cases. The presence of low wall shear stress was associated with recurrent events of endograft thrombosis, despite an otherwise normal anatomic and ultrasound assessment and a good distal run-off.

## Supplemental Material

sj-docx-1-jet-10.1177_15266028221091890 – Supplemental material for Computational Fluid Dynamics for the Prediction of Endograft Thrombosis in the Superficial Femoral Artery Click here for additional data file. Supplemental material, sj-docx-1-jet-10.1177_15266028221091890 for Computational Fluid Dynamics for the Prediction of Endograft Thrombosis in the Superficial Femoral Artery by Lennart van de Velde, Erik Groot Jebbink, Rob Hagmeijer, Michel Versluis and Michel M. P. J. Reijnen in Journal of Endovascular Therapy

## References

[1] Lammer J Zeller T Hausegger KA , et al. Sustained benefit at 2-years for covered stents versus bare-metal stents in long SFA lesions: the VIASTAR trial. Cardiovasc Intervent Radiol. 2015;38(1):25–32. doi:10.1007/s00270-014-1024-9. 25472936

[2] Reijnen MMPJ van Walraven LA Fritschy WM , et al. 1-year results of a multicenter randomized controlled trial comparing heparin-bonded endoluminal to femoropopliteal bypass. JACC Cardiovasc Interv. 2017;10(22):2320–2331. doi:10.1016/j.jcin.2017.09.013. 29169500

[3] Golchehr B Kruse R van Walraven LA , et al. Three-year outcome of the heparin-bonded Viabahn for superficial femoral artery occlusive disease. J Vasc Surg. 2015;62(4):984–989. doi:10.1016/j.jvs.2015.04.436. 26059093

[4] Lammer J Zeller T Hausegger KA , et al. Heparin-bonded covered stents versus bare-metal stents for complex femoropopliteal artery lesions: The randomized VIASTAR trial (viabahn endoprosthesis with propaten bioactive surface [VIA] versus bare nitinol stent in the treatment of long lesions in sup. J Am Coll Cardiol. 2013;62(15):1320–1327. doi:10.1016/j.jacc.2013.05.079. 23831445

[5] Golchehr B Holewijn S Kruse RR , et al. Efficacy of treatment of edge stenosis of endografts inserted for superficial femoral artery stenotic disease. Catheter Cardiovasc Interv. 2015;86(3):492–498. doi:10.1002/ccd.26061. 26103929

[6] Weinstock BS. Covered stents in the treatment of superficial femoral artery disease. Vasc Dis Manag. 2014;11(4):E76–E86.

[7] Gimbrone MA García-Cardeña G. Vascular endothelium, hemodynamics, and the pathobiology of atherosclerosis. Cardiovasc Pathol. 2013;22(1):9–15. doi:10.1016/j.carpath.2012.06.006. 22818581 PMC4564111

[8] Zilla P Bezuidenhout D Human P. Prosthetic vascular grafts: wrong models, wrong questions and no healing. Biomaterials. 2007;28(34):5009–5027. doi:10.1016/j.biomaterials.2007.07.017. 17688939

[9] Taylor CA Fonte TA Min JK. Computational fluid dynamics applied to cardiac computed tomography for noninvasive quantification of fractional flow reserve: scientific basis. J Am Coll Cardiol. 2013;61(22):2233–2241. doi:10.1016/j.jacc.2012.11.083. 23562923

[10] Gori T Polimeni A Indolfi C , et al. Predictors of stent thrombosis and their implications for clinical practice. Nat Rev Cardiol. 2019;16(4):243–256. doi:10.1038/s41569-018-0118-5. 30518952

[11] García Carrascal P García García J Sierra Pallares J , et al. Numerical study of blood clots influence on the flow pattern and platelet activation on a Stented Bifurcation Model. Ann Biomed Eng. 2017;45(5):1279–1291. doi:10.1007/s10439-016-1782-4. 28028712

[12] Ngoepe MN Frangi AF Byrne JV , et al. Thrombosis in cerebral aneurysms and the computational modeling thereof: a review. Front Physiol. 2018;9(306):1–22. doi:10.3389/fphys.2018.00306. 29670533 PMC5893827

[13] Zambrano BA Gharahi H Lim C , et al. Association of intraluminal thrombus, hemodynamic forces, and abdominal aortic aneurysm expansion using longitudinal CT Images. Ann Biomed Eng. 2016;44(5):1502–1514. doi:10.1007/s10439-015-1461-x. 26429788 PMC4826625

[14] Kabinejadian F Cui F Zhang Z , et al. A novel carotid covered stent design: in vitro evaluation of performance and influence on the blood flow regime at the carotid artery bifurcation. Ann Biomed Eng. 2013;41(9):1990–2002. doi:10.1007/s10439-013-0863-x. 23842696

[15] Ong CW Wee I Syn N , et al. Computational fluid dynamics modeling of hemodynamic parameters in the human diseased aorta: a systematic review. Ann Vasc Surg. 2020;63:336–381. doi:10.1016/j.avsg.2019.04.032. 31344467

[16] Tasso P Raptis A Matsagkas M , et al. Abdominal aortic aneurysm endovascular repair: profiling postimplantation morphometry and hemodynamics with image-based computational fluid dynamics. J Biomech Eng. 2018;140(11):111003. doi:10.1115/1.4040337. 30029263

[17] Conti M Ferrarini A Finotello A , et al. Patient-specific computational fluid dynamics of femoro-popliteal stent-graft thrombosis. Med Eng Phys. 2020;86:57–64. doi:10.1016/j.medengphy.2020.10.011. 33261734

[18] Antiga L Piccinelli M Botti L , et al. An image-based modeling framework for patient-specific computational hemodynamics. Med Biol Eng Comput. 2008;46(11):1097–1112. doi:10.1007/s11517-008-0420-1. 19002516

[19] Bijari PB Antiga L Wasserman BA , et al. Scan-Rescan reproducibility of carotid bifurcation geometry from routine contrast-enhanced MR angiography. J Magn Reson Imaging. 2011;33(2):482–489. doi:10.1002/jmri.22440. 21274992 PMC3059724

[20] Berg P VoÃŸ S Saalfeld S , et al. Multiple Aneurysms AnaTomy CHallenge 2018 (MATCH): phase I: segmentation. Cardiovasc Eng Technol. 2018;9(4):565–581. doi:10.1007/s13239-018-00376-0. 30191538

[21] McGah PM Nerva JD Morton RP , et al. In vitro validation of endovascular Doppler-derived flow rates in models of the cerebral circulation. Physiol Meas. 2015;36(11):2301–2317. doi:10.1088/0967-3334/36/11/2301. 26450643 PMC4684705

[22] Updegrove A Wilson NM Merkow J , et al. SimVascular: an open source pipeline for cardiovascular simulation. Ann Biomed Eng. 2017;45(3):525–541. doi:10.1007/s10439-016-1762-8. 27933407 PMC6546171

[23] Kung E Kahn AM Burns JC , et al. In vitro validation of patient-specific hemodynamic simulations in coronary aneurysms caused by Kawasaki disease. Cardiovasc Eng Technol. 2014;5(2):189–201. doi:10.1007/s13239-014-0184-8. 25050140 PMC4103185

[24] Chiu J-J Chien S. Effects of disturbed flow on vascular endothelium: pathophysiological basis and clinical perspectives. Physiol Rev. 2014;91(1). doi:10.1152/physrev.00047.2009.Effects. PMC3844671 21248169

[25] Jiménez JM Prasad V Yu MD , et al. Macro- and microscale variables regulate stent haemodynamics, fibrin deposition and thrombomodulin expression. J R Soc Interface. 2014;11(94). doi:10.1098/rsif.2013.1079. PMC3973357 24554575

[26] Javadzadegan A Lotfi A Simmons A , et al. Haemodynamic analysis of femoral artery bifurcation models under different physiological flow waveforms. Comput Methods Biomech Biomed Engin. 2015;19(11):1143–1153. doi:10.1080/10255842.2015.1113406 26582544

[27] Hathcock JJ. Flow effects on coagulation and thrombosis. Arterioscler Thromb Vasc Biol. 2006;26(8):1729–1737. doi:10.1161/01.ATV.0000229658.76797.30. 16741150

[28] Marin ML Veith FJ Cynamon J , et al. Human transluminally placed endovascular stented grafts: preliminary histopathologic analysis of healing grafts in aortoiliac and femoral artery occlusive disease. J Vasc Surg. 1995;21(4):595–603discussion603. doi:10.1016/S0741-5214(95)70191-5. 7535869

[29] Heyligers JM Verhagen HJ Rotmans JI , et al. Heparin immobilization reduces thrombogenicity of small-caliber expanded polytetrafluoroethylene grafts. J Vasc Surg. 2006;43(3):587–591. doi:10.1016/j.jvs.2005.10.038. 16520178

[30] Kang WY Campia U Bernardo NL , et al. How should we manage thrombosis of Viabahn stent-graft? A case report focused on catheter-directed thrombolysis. Cardiovasc Revasc Med. 2016;17(2):134–137. doi:10.1016/j.carrev.2016.01.011. 26994746

[31] Corbett SC Ajdari A Coskun AU , et al. In vitro and computational thrombosis on artificial surfaces with shear stress. Artif Organs. 2010;34(7):561–569. doi:10.1111/j.1525-1594.2009.00930.x. 20497159

[32] Stone HA Stroock AD Ajdari A. Engineering flows in small devices: microfluidics toward a lab-on-a-chip. Annu Rev Fluid Mech. 2004;36:381–411. doi:10.1146/annurev.fluid.36.050802.122124.

[33] Jensen JA Nikolov SI Yu AC , et al. Ultrasound vector flow imaging-part II: parallel systems. IEEE Trans Ultrason Ferroelectr Freq Control. 2016;63(11):1722–1732. doi:10.1109/TUFFC.2016.2598180. 27824556

[34] Campbell IC Ries J Dhawan SS , et al. Effect of inlet velocity profiles on patient-specific computational fluid dynamics simulations of the carotid bifurcation. J Biomech Eng. 2012;134(5). doi:10.1115/1.4006681. PMC3625536 22757489

[35] Morbiducci U Gallo D Massai D , et al. On the importance of blood rheology for bulk flow in hemodynamic models of the carotid bifurcation. J Biomech. 2011;44(13):2427–2438. doi:10.1016/j.jbiomech.2011.06.028. 21752380

[36] Lee SW Steinman DA. On the relative importance of rheology for image-based CFD models of the carotid bifurcation. J Biomech Eng. 2007;129(2):273–278. doi:10.1115/1.2540836. 17408332

[37] Torii R Wood NB Hadjiloizou N , et al. Fluid–structure interaction analysis of a patient-specific right coronary artery with physiological velocity and pressure waveforms. Commun Numer Methods Eng. 2009;25:565–580. doi:10.1002/cnm.1231.

[38] Al-Hakim R Lee EW Kee ST , et al. Hemodynamic analysis of edge stenosis in peripheral artery stent grafts. Diagn Interv Imaging. 2017;98(10):729–735. doi:10.1016/j.diii.2017.01.011. 28233711

[39] Arzani A Gambaruto AM Chen G , et al. Wall shear stress exposure time: a Lagrangian measure of near-wall stagnation and concentration in cardiovascular flows. Biomech Model Mechanobiol. 2017;16(3):787–803. doi:10.1007/s10237-016-0853-7. 27858174

