# Supplementary material for: Computational Fluid Dynamics for the Prediction of Endograft Thrombosis in the Superficial Femoral Artery
Source: J Endovasc Ther. 2022 Apr 25;30(4):615–27. doi: 10.1177/15266028221091890 (PMC10350734; doi:10.1177/15266028221091890)
Supplement: sj-docx-1-jet-10.1177_15266028221091890 – Supplemental material for Computational Fluid Dynamics for the Prediction of Endograft Thrombosis in the Superficial Femoral Artery [file sj-docx-1-jet-10.1177_15266028221091890.docx]

**SUPPLEMENTARY MATERIAL**

**Expanded Methods**

This supplement discusses details and limitations regarding the segmentation method, the derivation of the boundary conditions, the computational mesh as well as the simulation settings applied for the CFD solver. The overall modeling approach is illustrated in Supplementary Fig. 1.

The computational mesh was generated with SimVascular’s Tetgen mesher^1^ and was composed of tetrahedral elements and three layers of prismatic elements along the walls, with locally refined elements in a spherical region around the femoral bifurcation (twice as many tetrahedrals per volume). The numerical solution was considered mesh-converged if area-averaged WSS for steady peak flow at the first 5 cm of the proximal SFA wall changed no more than 5% when the number of mesh elements was doubled. The number of elements in the converged mesh is shown in Supplementary Table 1.

For the simulations, the open-source finite-element SimVascular solver^2^ (version 2017-08-14) tailored for personalized blood flow simulations was used with backflow stabilization^3^ and RCR outflow boundary conditions^4^. The vessel wall was considered rigid and blood was approximated as a Newtonian fluid. Previous simulations in bifurcations of similar size have shown that the rigid wall assumption^5^ and the Newtonian-fluid assumption^6,7^ can impact the absolute values of WSS by 5-10%, but do not change the spatial distribution of low and high WSS. These assumptions are therefore unlikely to alter the implications of the CFD results. The density of blood was set at 1059 kg.m^-3^ ^8^, and blood viscosity was estimated from the patient’s measured hematocrit value (0.3-0.43) a few weeks before stent occlusion by Batchelor’s formula ^9^

$\mu_{blood}=\mu_{plasma}(1+2.5\epsilon+6.2\epsilon^{2})$,

with $\mu_{plasma}$ equal to 1.2 mPa.s and $\epsilon$ the volumetric cell fraction of blood, considered equal to the hematocrit value.

For the implicit time discretization method, the timestep was set to be below the estimated Courant-Friedrichs-Lewy number ^10^, given by

$$CFL=\frac{v\Delta t}{h}$$

with *v* the velocity*, t* the timestep and *h* the element edge length. The values were estimated at a location where maximum velocity was expected, at which the velocity was estimated by taking the peak flow rate and assuming a parabolic flow profile. Timesteps were checked for residual convergence (a reduction of the residual of at least three orders), which was achieved for each timestep in the four simulated cases. Starting from the steady-state solution for the mean flow rate, five cardiac cycles were simulated, during which a periodic solution was reached for all cases. The last cycle was used for analysis. Time-averaged wall shear stress for this cycle was evaluated as the shear component of the traction vector by

$\text{TAWSS}=\frac{1}{T}\int_{0}^{T}\left| \boldsymbol{\sigma n}-\left( \boldsymbol{\sigma n}\cdot\boldsymbol{n} \right)\boldsymbol{n} \right|d$t,

with $T$ the cycle period, $\boldsymbol{\sigma}$ the Cauchy stress tensor at the wall, $\boldsymbol{n}$ the unit vector normal to the wall, and $t$ time.

**References**

1. Si H. TetGen, a Delaunay-Based Quality Tetrahedral Mesh Generator. *ACM Trans Math Softw*. 2015;41(2):1-36. doi:10.1145/2629697

2. Updegrove A, Wilson NM, Merkow J, Lan H, Marsden AL, Shadden SC. SimVascular: An Open Source Pipeline for Cardiovascular Simulation. *Ann Biomed Eng*. 2017;45(3):525-541. doi:10.1007/s10439-016-1762-8

3. Esmaily Moghadam M, Bazilevs Y, Hsia TY, Vignon-Clementel IE, Marsden AL. A comparison of outlet boundary treatments for prevention of backflow divergence with relevance to blood flow simulations. *Comput Mech*. 2011;48(3):277-291. doi:10.1007/s00466-011-0599-0

4. Vignon-Clementel IE, Figueroa CA, Jansen KE, Taylor CA. Outflow boundary conditions for 3D simulations of non-periodic blood flow and pressure fields in deformable arteries. *Comput Methods Biomech Biomed Engin*. 2010;13(5):625-640. doi:10.1080/10255840903413565

5. Torii R, Wood NB, Hadjiloizou N, et al. Fluid–structure interaction analysis of a patient-specific right coronary artery with physiological velocity and pressure waveforms. *Commun Numer methods Eng*. 2009;25:565-580. doi:10.1002/cnm.1231

6. Lee S-W, Steinman DA. On the Relative Importance of Rheology for Image-Based CFD Models of the Carotid Bifurcation. *J Biomech Eng*. 2007;129(2):273-278. doi:10.1115/1.2540836

7. Morbiducci U, Gallo D, Massai D, et al. On the importance of blood rheology for bulk flow in hemodynamic models of the carotid bifurcation. *J Biomech*. 2011;44(13):2427-2438. doi:10.1016/j.jbiomech.2011.06.028

8. Trudnowski RJ, Rico RC. Specific gravity of blood and plasma at 4 and 37 °C. *Clin Chem*. 1974;20(5):615-616. doi:10.1093/clinchem/20.5.615

9. Yilmaz F, Gundogdu MY. A critical review on blood flow in large arteries; relevance to blood rheology, viscosity models and physiologic conditions. *Kore-Australia Rheol J*. 2008;20(4):197-211.

10. Courand PY, Dinic M, Lorthioir A, et al. Resistant hypertension and atherosclerotic renal artery stenosis effects of angioplasty on ambulatory blood pressure. A retrospective uncontrolled single-center study. *Hypertension*. 2019;74(6):1516-1523. doi:10.1161/HYPERTENSIONAHA.119.13393

**
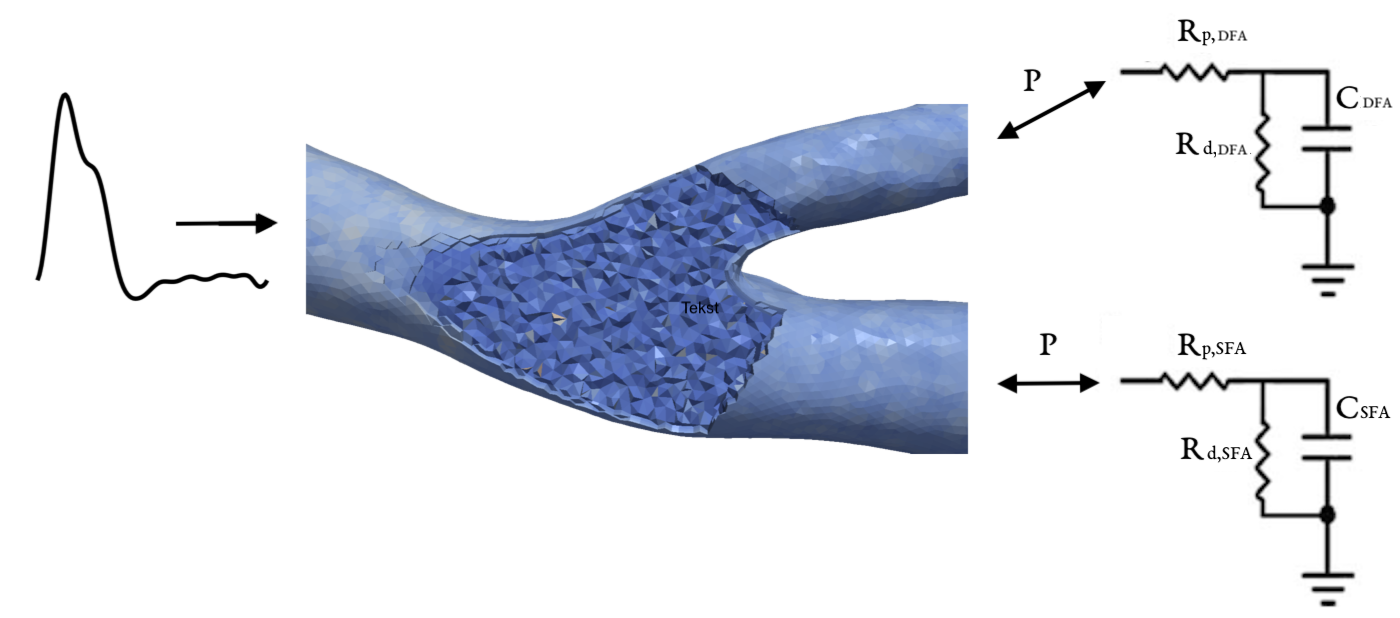
Supplementary Figure 1**

Modeling overview for the boundary conditions applied for the simulations. A time-varying flow rate is specified at the inlet (CFA) and an integral pressure-flow relationship is enforced at the outlets (SFA and DFA), through RCR-models tuned to reproduce measured flow rates in the distal vasculature. In addition, part of the final volumetric CFD mesh is shown in the center. DFA: deep femoral artery, SFA: superficial femoral artery.

**Supplementary Table 1 – CFD simulation parameters**

|  | Case 1 | Case 2 | Case 3 | Case 4 |
| --- | --- | --- | --- | --- |
| Number of mesh elements | 1,063,029 | 831,614 | ﻿1,184,166 | 867,626 |
| SFA Windkessel |  |  |  |  |
| R_p_ (kg.mm^-4^s^-1^) | $6.47\times{10}^{-4}$ | $3.82\times{10}^{-5}$ | $1.45\times{10}^{-4}$ | $6.48\times{10}^{-5}$ |
| C (mm^4^s^2^kg^-1^) | 246 | 955 | 266 | 381 |
| R_d_ (kg.mm^-4^s^-1^) | $7.2\times{10}^{-3}$ | $2.6\times{10}^{-3}$ | $4.83\times{10}^{-3}$ | $6.99\times{10}^{-3}$ |
| DFA Windkessel |  |  |  |  |
| R_p_ (kg.mm^-4^s^-1^) | $2.39\times{10}^{-5}$ | $2.39\times{10}^{-5}$ | $2.39\times{10}^{-5}$ | $1.30\times{10}^{-5}$ |
| C (mm^4^s^2^kg^-1^) | 123 | 1.36 $\times{10}^{3}$ | 296 | 254 |
| R_d_ (kg.mm^-4^s^-1^) | $1.08\times{10}^{-2}$ | $1.89\times{10}^{-3}$ | $4.7\times{10}^{-3}$ | $9.0\times{10}^{-3}$ |
| Pressure range (mmHg) | 65.2 – 99.1 | 70.6 – 124.3 | 83.7 – 121.4 | 77.2 – 114.9 |

C = Windkessel compliance, R_p_ = proximal Windkessel resistance, R_d_ = peripheral Windkessel resistance

﻿﻿﻿﻿﻿﻿
